# Centrifugal Force-Spinning to Obtain Multifunctional Fibers of PLA Reinforced with Functionalized Silver Nanoparticles

**DOI:** 10.3390/polym15051240

**Published:** 2023-02-28

**Authors:** María Dolores Martín-Alonso, Valentina Salaris, Adrián Leonés, Víctor Hevilla, Alexandra Muñoz-Bonilla, Coro Echeverría, Marta Fernández-García, Laura Peponi, Daniel López

**Affiliations:** 1 Instituto de Ciencia y Tecnología de Polímeros (ICTP-CSIC), Calle Juan de la Cierva 2, 28006 Madrid, Spain; 2 IMDEA Materials Institute, Calle Eric Kandel 2, 28906 Getafe, Spain

**Keywords:** centrifugal force-spinning, PLA, nano/micro fibers, biopolymers, nanoparticles, nanocomposites

## Abstract

The design and development of multifunctional fibers awakened great interest in biomaterials and food packaging materials. One way to achieve these materials is by incorporating functionalized nanoparticles into matrices obtained by spinning techniques. Here, a procedure for obtaining functionalized silver nanoparticles through a green protocol, using chitosan as a reducing agent, was implemented. These nanoparticles were incorporated into PLA solutions to study the production of multifunctional polymeric fibers by centrifugal force-spinning. Multifunctional PLA-based microfibers were obtained with nanoparticle concentrations varying from 0 to 3.5 wt%. The effect of the incorporation of nanoparticles and the method of preparation of the fibers on the morphology, thermomechanical properties, biodisintegration, and antimicrobial behavior, was investigated. The best balance in terms of thermomechanical behavior was obtained for the lowest amount of nanoparticles, that is 1 wt%. Furthermore, functionalized silver nanoparticles confer antibacterial activity to the PLA fibers, with a percentage of killing bacteria between 65 and 90%. All the samples turned out to be disintegrable under composting conditions. Additionally, the suitability of the centrifugal force-spinning technique for producing shape-memory fiber mats was tested. Results demonstrate that with 2 wt% of nanoparticles a good thermally activated shape-memory effect, with high values of fixity and recovery ratios, is obtained. The results obtained show interesting properties of the nanocomposites to be applied as biomaterials.

## 1. Introduction

Nowadays, the growing interest in multifunctional polymeric-based materials is a key factor to be focused on. Among them, shape-memory polymers (SMPs) play an important role. In particular, shape-memory polymers are a class of smart materials able to recover their original shape from a previously temporary programmed shape when exposed to external stimuli of different nature such as temperature, moisture, pH, light, etc. [1,2,3,4]. Therefore, the shape-memory effect (SME) results from a combination of polymer chemistry, morphology, and a specific processing condition. SMPs possess tremendous potential in many fields as minimally invasive medical devices, actuators, sensors, and smart textiles, among others [5,6,7,8,9]. Depending on the processing of the materials and of their final structures, different types of shape-memory blocks, shape-memory foams, shape-memory fibers, and shape-memory films can be obtained [10,11,12]. Recently, SMPs with fibrous structures are gaining interest in applications that imply contact with the human body such as smart textiles or scaffolds for regenerative medicine. In this sense, two main factors have to be considered, that is, on the one hand, the obtention of polymeric fibrous structures, and the use of biocompatible and or biodegradable polymers, on the other hand [13,14]. In this regard, the electrospinning process has been recognized as one of the most attractive technologies to produce fibrous structures, thanks to the advantageous high porosity and specific surface area obtained [14,15,16,17]. The electrospinning process is a simple, extremely versatile, and low-cost process for obtaining polymeric fibers recently scaled up at the industrial level, that can find several applications in biomedicine but also in food packaging and agriculture [18,19,20,21,22,23,24]. In fact, by solution electrospinning, it is possible to produce multifunctional thin polymeric materials in the form of woven non-woven fibers mats from polymeric solutions subjected to high electric voltage and at room temperature [25,26]. Electrospinning is currently the most popular method for producing polymer nano and microfibers. However, high voltage, which is sensitive to the dielectric constant, safety concerns, and low fiber yields limit the application of electrospinning [27]. The search for a method that would minimize, or even eliminate, many of the limitations encountered in electrospinning is focused on increasing material choice, improving production rate, and lowering fiber costs through an environmentally friendly process. In the centrifugal force-spinning method, the electric field, used in the electrospinning process, is replaced by centrifugal forces [28,29]. The combination of centrifugal forces with multiple configurations of easily interchangeable spinnerets makes centrifugal force-spinning a versatile method that overcomes many of the limitations of existing processes, namely, high electric fields and a solution that is typically dielectric. These changes significantly increase the selection of materials by allowing both non-conductive and conductive solutions to be spun into nanofibers [28,30,31]. If necessary, a high-temperature solvent can also be used by heating the spinneret holding the material of interest. Additionally, solid materials can be melted and spun without the need for chemical preparation and, therefore, for solvent recovery since no solvent is involved in the process [32].

Among polymers frequently used in solution electrospinning, poly(lactic acid), PLA, a non-petrochemical biodegradable polymer, is considered of high interest for food packaging applications as well as for the biomedicine field. PLA shows high tensile strength and elastic modulus, biocompatibility, and degradability under physiological conditions into non-toxic products, making of PLA an ideal material for practical use in contact with the human body. This versatile polymer is known to exhibit a thermo-responsive shape-memory effect, activated by its glass transition temperature (T_g_) acting as transition temperature, around 60 °C [13,33,34]. However, its T_g_ is much higher than the human body temperature, together with its brittleness, poor toughness, and elongation at break < 10%, which limits its direct use in biomedical applications. Thermally-activated shape-memory effect, in general, and, in particular, in PLA is achieved by heating up to a switching temperature (T_sw_) higher than its T_g_ in the case of PLA, at which polymer chains have enough mobility to recover the original form from a temporary shape previously fixed. Thus, to consider the thermally-activated shape-memory effect of PLA as a suitable way to change the temporary form of devices for biomedical applications, a T_sw_ closer to the human body has to be achieved [13,35].

Moreover, unique mechanical, electrical, chemical, and optical properties have been achieved by modifying polymers, fibers or not, with the addition of nanoparticles (NPs), obtaining polymeric nanocomposites with both enhanced and functional properties just compared with the neat matrix. Amounts, size, and proper distribution of NPs into the polymer matrix can effectively control the properties of these nanocomposites [5]. Recently, the utilization of polymers with embedded silver (Ag) NPs have attracted much attention mainly due to their antimicrobial activities [36]. Silver NPs show antibacterial activity toward germs on contact without the release of toxic biocides. Therefore, the antimicrobial properties of Ag NPs can be considered non-toxic and environmentally friendly materials in biomedical applications. The Ag-NPs-filled polymeric materials have to release the Ag ions to a pathogenic environment continuously in order to be efficacious. Therefore, silver nanoparticles gain attention due to their remarkable and unique optical, mechanical, and catalytic properties in addition to their conductivity, heat transfer, and many other excellent applications in biomedical fields. Moreover, Ag NPs have been approved for use in food-contact polymers in the USA and the European Union, after previous consideration of the use conditions [37,38]. Consequently, the development of stable NPs is presented in a wide range of applications in the field of biomedical science. Inorganic noble metal NPs are proven for their antimicrobial properties [37,38,39].

NPs can be synthesized by different physicochemical methods and they need to reach an optimal dispersion into the polymeric matrix, obtained by modifying them through physical or chemical functionalization. Recently, attention has been paid to the synthesis under eco-friendly conditions of silver and gold (Au) nanoparticles [37] by using chitosan instead of sodium borohydride, NaBH_4_, as reducing agent and citrate, or ascorbate, which are associated with environmental toxicity or biological hazards. Chitosan (CH), is one of the well-known and most used biopolymers, derivative of chitin. In fact, thanks to their large amount of free amino and hydroxyl groups, under the proper thermal conditions, they can be used as reducing and stabilizing agents for the synthesis of Ag and Au nanoparticles, preventing also their aggregation [37,39,40,41]. This fact is possible, thus, considering that in proper thermal conditions, as previously reported in the literature, the hydroxyl groups can be converted into carboxyl groups by air oxidation, which reduces the silver ions [37].

The synthesis of nanoparticles from chitosan has been paid great attention to due to their biocompatibility, biodegradability, and hydrophilic properties, which endow them with opportunities for various applications [37]. The polymeric feature of chitosan interacts with negatively-charged molecules and polymers. Due to the interaction that takes place between the active amino groups in chitosan and metal nanoparticles, chitosan is chosen as a protecting agent in the synthesis of metal nanoparticles. Both active amino and hydroxyl functional groups in chitosan show many remarkable biological activities including antimicrobial activity for disease resistance toward a number of human cell types [41]. In this regard, chitosan can play the role of a reducing agent. The reduction of Ag+ ions is coupled to the oxidation of the hydroxyl groups in molecular CH and/or its hydrolyzates. The reducing ability of chitosan depends on its concentration and reaction temperature. Thus, by optimizing the experimental conditions such as temperature, Ag+, and CH concentration, monodisperse AgCH NPs with highly antibacterial and antiproliferative activities could be obtained. Moreover, it is interesting to note that, in this case, chitosan is able to act as both a reducing agent and stabilizer for the NPs. In fact, the excess of amine and hydroxyl groups present in the chitosan supports the nucleation as well as the stabilization of Ag NPs during the process. In other words, the Ag NPs adsorbed on the surface of the polymer are prevented from further aggregation [39,40,41,42].

With this background, in this study, eco-friendly synthesis conditioning to obtain functionalized silver nanoparticles with a green protocol, based on non-toxic biodegradable chitosan as a reducing agent, has been implemented to incorporate the nanoparticles into a PLA solution, with the final aim to study PLA-based reinforced fibers obtained by centrifugal force-spinning.

## 2. Materials and Methods

### 2.1. Materials

Polylactic acid (PLA3051D, 3% of D-lactic acid monomer, molecular weight 142 × 10^4^ g/mol, density 1.24 g/cm^3^) was supplied from NatureWorks^®^ ((NatureWorks LLC, Min- netonka, MN, USA). Silver nitrate (AgNO_3_) and chitosan from shrimp shells with a deacetylation degree > 75% were purchased from Sigma-Aldrich (St. Quentin Fallavier, France). Acetic acid and sodium hydroxide were purchased from Fluka (Seelze, Germany).

### 2.2. Synthesis of Based Chitosan Silver Nanoparticles

Chitosan-based silver nanoparticles (AgCH-NPs) were synthesized by a method described elsewhere [36]. Typically, 4.5 mL of a solution 52.0 mM of AgNO_3_ and 10 mL of a solution of chitosan of concentration 6.92 mg/mL in 1% acetic acid were mixed and heated at 95 °C under stirring for 12 h in an argon-purified reactor. The dispersions obtained were dialyzed for six days with distilled water using dialysis membranes with a MWCO of 6000–8000 Da [36].

### 2.3. Preparation of Poly(Lactic Acid) Forced-Fibers Containing AgCH-NPs

**Scheme 1:**
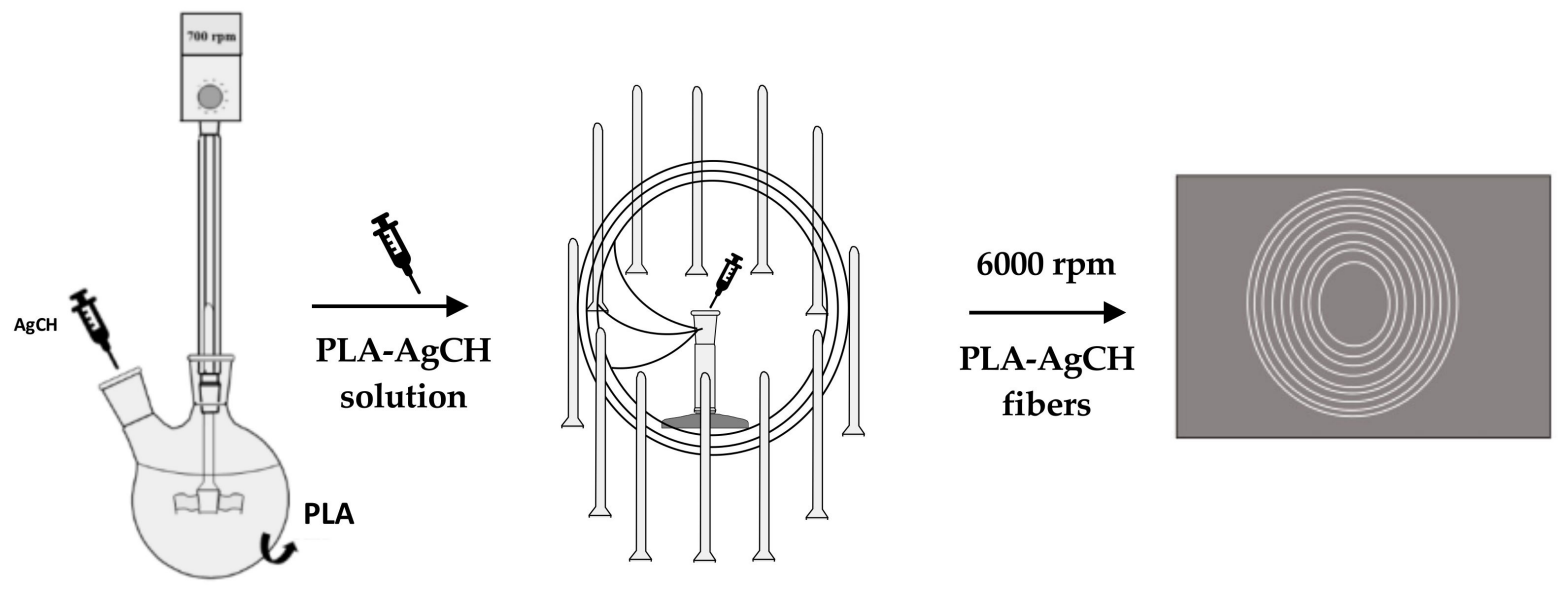
Methodology followed in the experimental procedure to obtain the f-fibers.

Experimental conditions for centrifugal force-spinning were established after conducting an exploratory study in which the concentration of the PLA solution was varied from 2 to 20 wt% and the rotation speed was varied between 6000 and 10,000 rpm, with a fixed distance spinneret-collector of 10 cm. The criteria for fixing the spinning parameters were based on the best results obtained in terms of fiber production, mesh homogeneity, and absence of defects. In this way, appropriate amounts of PLA and AgCH-NPs were mixed in a two-mouth flask with a shovel shaker at 700 rpm. PLA pellets were previously dried overnight at 60 °C, in order to avoid the presence of moisture. The final volume (PLA solution + NPs solution) was kept constant at 25 mL. Briefly, PLA solution was first prepared by mixing the polymer with the appropriate amount of CHCl_3_, in order to maintain a fixed concentration of PLA 12 %*w*/*v* in the final solution, by magnetic stirring. AgCH NPs dispersed in the appropriate amount of CHCl_3_ were added dropwise while the PLA solution was stirred with a shovel shaker at 300–400 rpm. Once the amount of nanoparticles was added, the solution was mixed for 20 min at 700 rpm of stirring to ensure the homogenous dispersion of NPs. Finally, these mixtures were taken to the force-spinning equipment (see Scheme 1) to produce forced-fibers, f-fibers. A total amount of 25 mL of the PLA/AgCH-NPs solution was injected into the needle-based spinneret (needle gauge of 0.4 mm). The solution was spun at 6500 rpm and collected as mats, which were wrapped into an aluminum foil (see Scheme 1). The spinneret-collector distance was kept constant at 10 cm.

### 2.4. Characterization Techniques

A Philips XL30 scanning electron microscope ((SEM, Phillips, Eindhoven, The Netherlands), with an accelerating voltage of 10 kV, work distance of 10–15 mm was used to record SEM micrographs of samples and observe changes produced by the different amount of nanofillers (AgCH-NPs). Each specimen was gold-coated (~5 nm thickness) in a Polaron SC7640 Auto/Manual Sputter (Quorum Technoligies, Newheaven, U.K.).

Images of nanoparticles were taken using a Field Emission Scanning electron microscopy (FE-SEM) Hitachi SU 8000 with an acceleration voltage of 30 kV.

UV-vis spectroscopy was performed on a Lamda 35 Perkin Elmer instrument (Perkin Elmer Spain, S.L., Madrid, Spain).

Fourier transform infrared (FTIR) spectra were recorded for all the samples using a Spectrum-One Perkin Elmer (Perkin Elmer Spain, S.L., Madrid, Spain) spectrometer between 650 and 4500 cm^−1^ spectral range with a 4 cm^−1^ resolution. A background spectrum was acquired before every sample and all samples were vacuum-dried prior to measurement.

Thermal transitions and stability of neat PLA as well as nanocomposites with different amounts of AgCH-NPs f-fibers, were studied by differential scanning calorimetry (DSC) and thermogravimetric analysis (TGA) in a DSC Q2000 and TA-Q500 apparatus both from TA Instruments (TA Instruments, New Castle, DE, USA), respectively. In the DSC, samples were heated from −60 °C up to 180 °C at 10 °C/min under a N_2_ atmosphere (flow rate of 50 mL/min). Glass transition temperatures (T_g_), calculated as the midpoint of the transition, melting temperatures (T_m_), and both cold crystallization (ΔH_cc_) and melting (ΔH_m_) enthalpies were calculated by analyzing the thermograms in the TA Universal Analysis software. The degree of crystallinity (X_c_%) was, therefore, obtained from Equation (1), using 93.6 J/g as the crystallization enthalpy value for pure crystalline PLA (ΔH_0m_) [43]. W_f_ represents the weight fraction of pure PLA present in the sample (W_f_ =1 − m_f_, where mf is the weight fraction of the nanoparticles in the nanocomposite):

(1) $$\mathrm{Xc}\;\%=\frac{\Delta \mathrm{Hm}\;-\;\Delta \mathrm{Hcc}}{\Delta H0m}\times \frac{1}{\mathrm{Wf}}\times 100$$

Tensile tests were carried out in an Instron instrument equipped with a 100 N load cell, operated at room temperature and at a crosshead speed of 10 mm/min. The initial length between clamps was set at 10 mm, samples of 5 mm width and ~80 μm of average thickness were measured and results from five to ten specimens were averaged. The Young’s modulus (slope of the curve from 0–2% deformation), maximum stress, ultimate tensile strength, and elongation at break were calculated.

Thermally-activated shape-memory characterization has been performed by dynamic mechanical thermal analysis (DMTA) in a DMA Q800 TA instruments (TA Instruments, New Castle, DE, USA), by using thermo-mechanical cyclic tests. The instrument was set in controlled force mode and four different stages were defined for each cycle:

- the sample was equilibrated at the chosen switching temperature (T_sw_) for 5 min, in this case, the T_g_ of PLA matrix, 60 °C;
- ramp stress of 0.2 MPa min^−1^ was applied until the sample reached 50% of deformation;
- the sample was subsequently cooled at a fixing temperature (T_fix_) of 10 °C under constant stress in order to fix the temporary shape;
- after releasing the stress at 0.50 MPa min^−1^, the sample was heated at 3 °C min^−1^ up to T_sw_ and maintained for 30 min in order to recover the initial permanent shape.

Sample dimensions for the DMTA and shape-memory tests were the same as for the previously described mechanical tests. The quantification of the shape-memory behavior was carried out by calculating the strain fixity ratio (R_f_) and the strain recovery ratio (R_r_) given by Equations (2) and (3), respectively [13,44]:

(2) $$R_{f}\left(N\right)=100\times \frac{{ɛ}_{u}\left(N\right)}{{ɛ}_{m}\;\left(N\right)}$$

(3) $$R_{r}\left(N\right)=100\times \frac{{ɛ}_{m}\left(N\right)-{ɛ}_{p}\left(N\right)}{{ɛ}_{m}\left(N\right)-{ɛ}_{p}\left(N-1\right)}$$

where ɛ_m_ is the maximum strain after cooling to T_fix_ and before releasing the stress, ɛ_u_ is the fixed strain after releasing the stress at T_fix_ and ɛ_p_ is the residual strain after retaining the sample at T_sw_ for 30 min. In particular, R_f_ indicates the ability of the material to fix the temporary shape, and R_r_, its capability to recover its original shape.

To study the crystalline structure of the plasticized PLA nanocomposite films, a Bruker D8 Advance X-ray diffractometer (Bruker, Madrid, Spain) equipped with a CuKα (λ = 0.154 nm) source was used. Samples were mounted on an appropriate holder and scanned between 2° and 60° (2θ) with a scanning step of 0.02°, a collection time of 10 s per step, and 40 kV of operating voltage.

Antimicrobial activity of the prepared nanocomposites was determined following the E2149-13a standard method of the American Society for Testing and Materials (ASTM) [45] against *Staphylococcus aureus* (*S. aureus*, ATCC 29213) bacteria. Each nanocomposite was placed in a sterile falcon tube containing bacterial suspension (ca. 10^4^ colony forming units (CFU)/mL in phosphate-buffered saline, pH 7.4). Falcon tubes with only the inoculum and neat PLA were also prepared as control experiments. The samples were shaken at room temperature at 150 rpm for 24 h. Bacterial concentrations at time 0 and after 24 h were calculated by the plate count method. The measurements were made at least in triplicate.

The biodegradation test for all the samples was conducted under aerobic composting conditions in a laboratory-scale reactor following the ISO-20200 standard [46,47]. Briefly, samples were cut into square geometries of 15 mm × 15 mm and buried 4–6 cm in depth inside reactors containing solid biodegradation media 10 wt% of compost (Compo, Spain), 30 wt% of rabbit food, 10 wt% of starch, 5 wt% of sugar, 1 wt% of urea, 4 wt% of corn oil, 40 wt% of sawdust and water in a 45:55 wt% ratio) and incubated at 58 °C for 30 days. Samples were kept in textile meshes to allow easy removal from the composting medium, while when buried access to microorganisms and moisture was ensured. Water was periodically added to the reaction containers in order to maintain the relative humidity in the medium, and the aerobic conditions were guaranteed by regular mixing of the compost medium. Samples were recovered from the disintegration medium at different time intervals (7, 17, 21, 28, 36, and 44 days), cleaned carefully, and dried in an oven at 37 °C until constant weight. The mass loss weight % was calculated by normalizing the sample weight at different incubation times to the initial weight value. Photographs were taken from samples once extracted from the composting medium.

## 3. Results and Discussion

**Figure 1:**
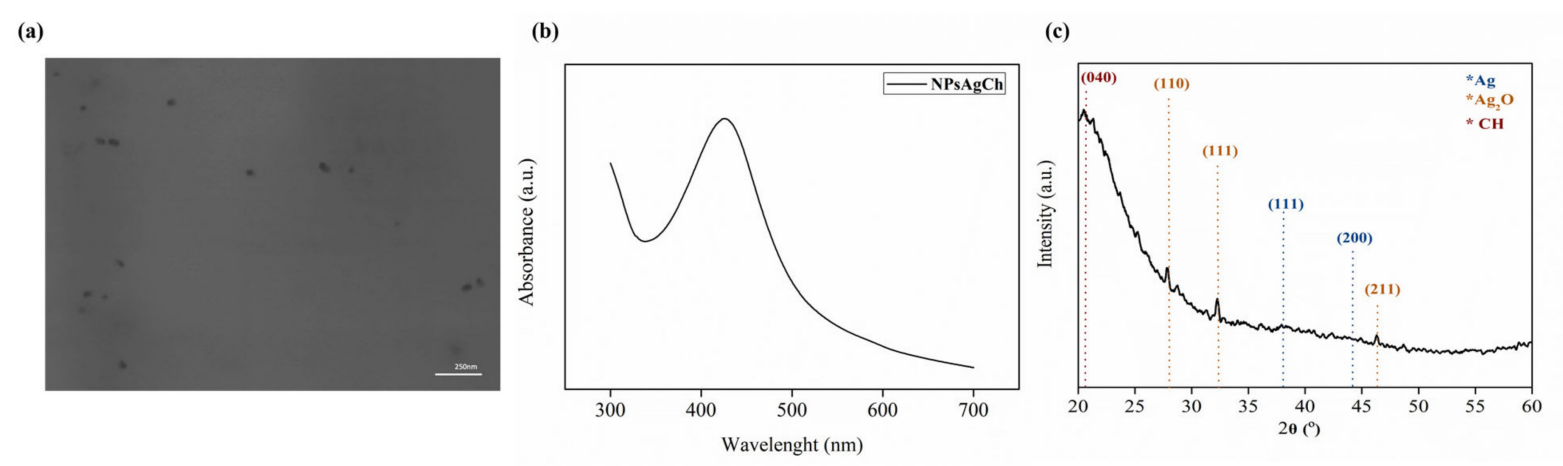
(**a**) FE-SEM micrograph at 100,000 magnification of chitosan-based silver nanoparticles (AgCH-NPs) prepared by reducing AgNO_3_ in a chitosan solution. (**b**) UV–Vis absorption spectra of chitosan-based silver nanoparticles (AgCH-NPs). (**c**) XRD spectrum of synthesized AgCH-NPs.

Firstly, the synthesis of AgCH-NPs was carried out and confirmed by FE-SEM, UV-Vis analysis as well as by crystallography analysis, as reported in Figure 1a–c, respectively. In particular, the morphology of AgCH-NPs was studied by FE-SEM. Figure 1a shows a representative microphotograph, which reveals a spherical morphology with an average diameter of ~8 ± 1 nm. This result agrees with previously reported values for samples obtained by the same procedure [48]. In addition, from the UV–Vis analysis, the characteristic surface plasmon resonance of silver, centered in 420 nm is obtained by studying the AgCH-NPs solution after lyophilization of the reduced solution of silver nitrate with chitosan [49].

Meanwhile, in Figure 1b, XRD has been used in order to identify the characteristic peaks at 38° and 44°, which indicates face-centered cubic Ag crystals, the presence of chitosan at about 20° and the peaks at about 28°, 32° and 46° which correspond to the plane (110), (111) and (211) of Ag_2_O [50].

In fact, it is known that chitosan always contains bound water (~5%) even when it has been dried to the extreme. The incorporation of bound water into the crystal lattice, commonly termed hydrated crystals, generally gives rise to a more dominated polymorph which can be detected by a broad crystalline peak in the corresponding X-ray pattern. The peak registered near 20.2° is reported to be the indication of the relatively regular crystal lattice (040) of chitosan [51].

Therefore, once the functionalized nanoparticles are obtained, different concentrations of them have been used to be dispersed into a PLA solution, as indicated in Table 1, in order to obtain the reinforced forced-fibers, f-fibers, with different formulations.

**Figure 2:**
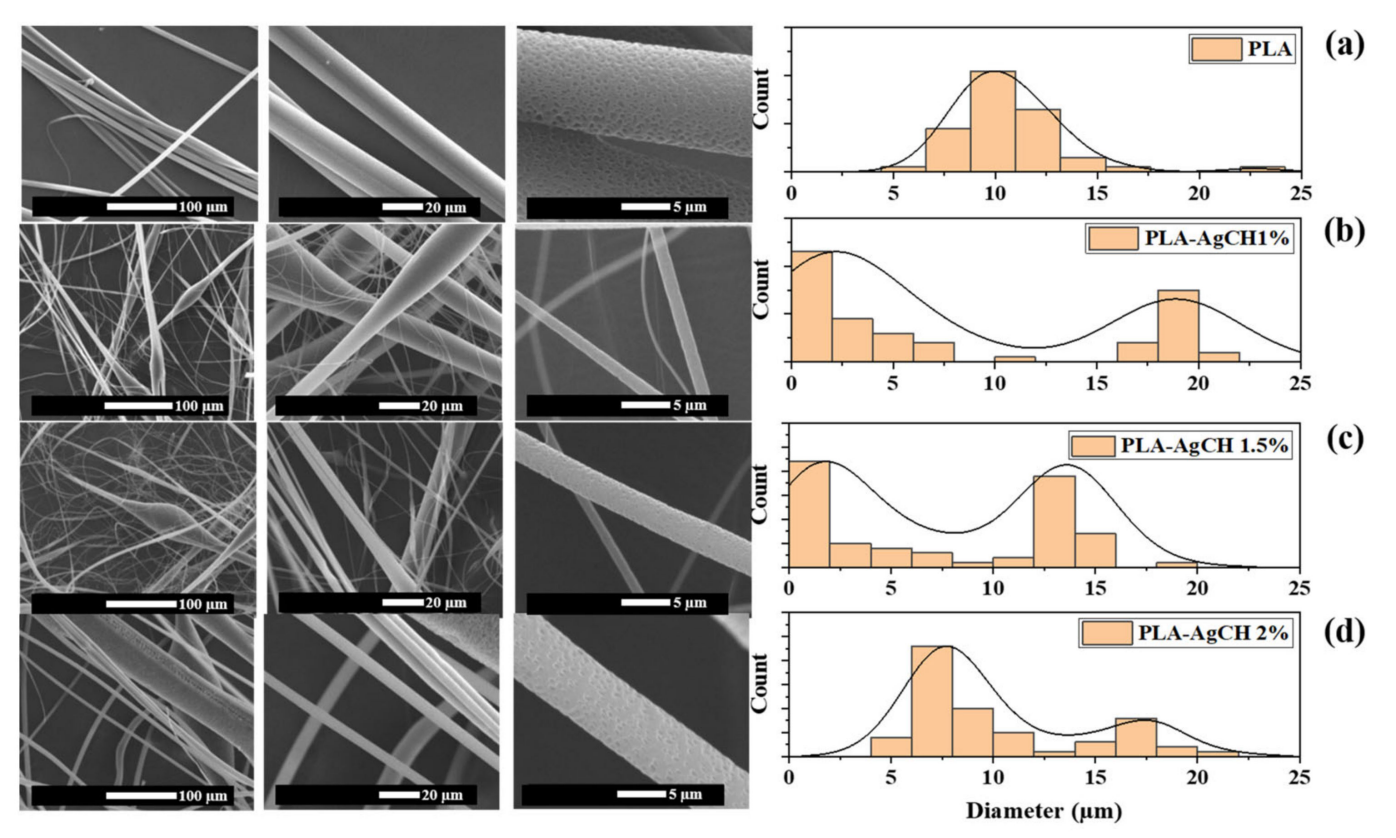
SEM images of PLA and PLA-AgCH-NPs reinforced f-fibers at different magnification, with their corresponding diameter distribution. (**a**) PLA, (**b**) 1 wt% AgCH-NPs, (**c**) 1.5 wt% AgCH-NPs, (**d**) 2 wt% AgCH-NPs.

**Figure 3:**
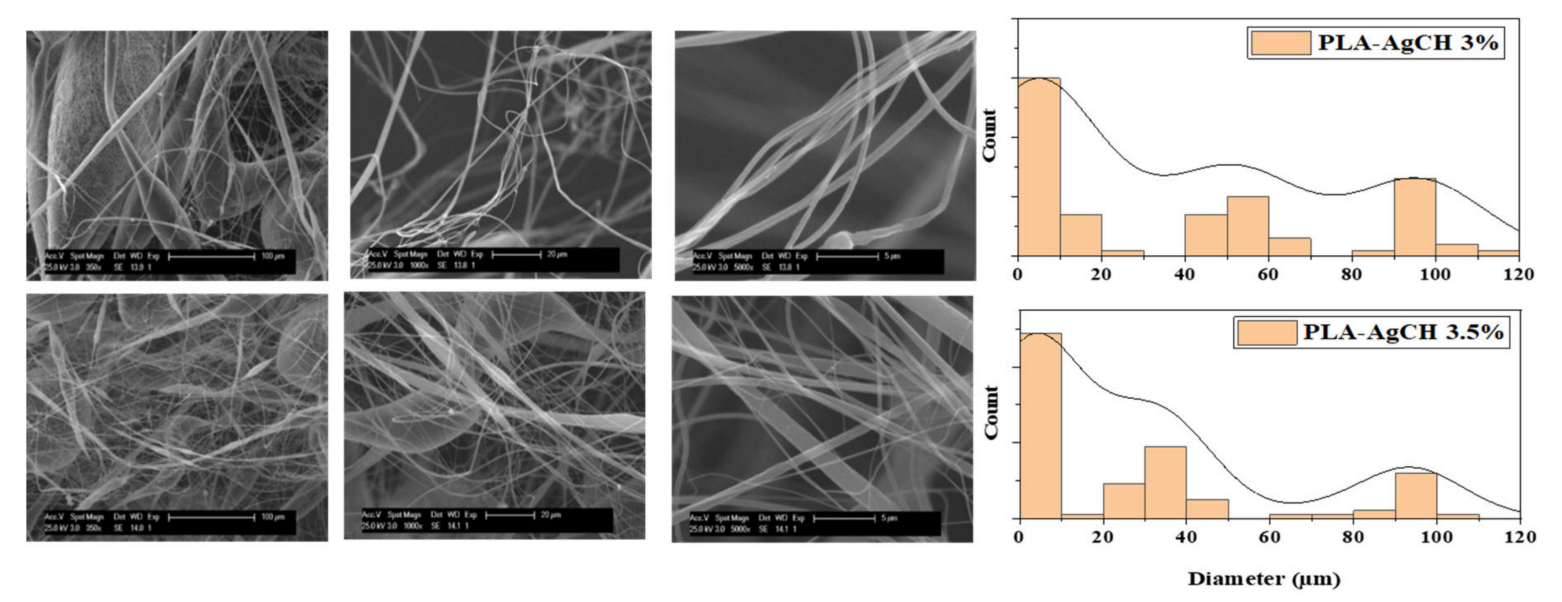
SEM images of PLA-AgCH-NPs reinforced f-fibers at different magnification, with their corresponding diameter distribution for high amount of NPS, that is 3 and 3.5 wt% AgCH-NPs.

Once the f-fibers based on PLA reinforced with different concentrations of AgCH-NPs were obtained, their morphology was studied by scanning electron microscopy (SEM) as reported in Figure 2 and Figure 3 for the different concentrations of NPs. In general, for each case, it is possible to observe that, by adding NPs, the fiber morphology changes, resulting in fibers with an increasing number of defects and lower homogeneity in size. In particular, the distribution of f-fibers diameters can be centered into different zones depending on the amount of NPs. For the neat PLA f-fibers, the diameter distribution is centered on a single point with an average value of about 10 μm, while for f-fibers reinforced with small amounts of NPs, that is 1, 1.5, and 2 wt%, the diameter distribution obtained presents a bimodal distribution (Figure 2), with one of them centered at low diameter values, less than 5 µm, and the other at larger ones between 10 and 20 µm, increased with an increasing amount of NPs.

Different behavior can be found when a high amount of NPs, such as 3 and 3.5 wt% have been added to the PLA f-fibers, as reported in Figure 3. In fact, the images reflect a material more heterogeneous, with a big variation on the diameter distribution, in respect to the others. In particular, in these f-fibers, a new distribution zone is obtained at higher diameters, centered at about 90 µm.

This phenomenon can be explained due to a critical reinforced effect in PLA when 3 wt% or more AgCH-NPs were added, where the solution cannot be spun correctly, producing fiber aggregates of ~90 µm in diameter. In fact, a significative change in the morphology of the reinforced f-fibers is evidenced between PLA reinforced with a low amount of NPs, PLA-AgCH 1/1.5/2 wt%, and PLA reinforced with a high amount of NPs PLA-AgCH 3/3.5%.

**Figure 4:**
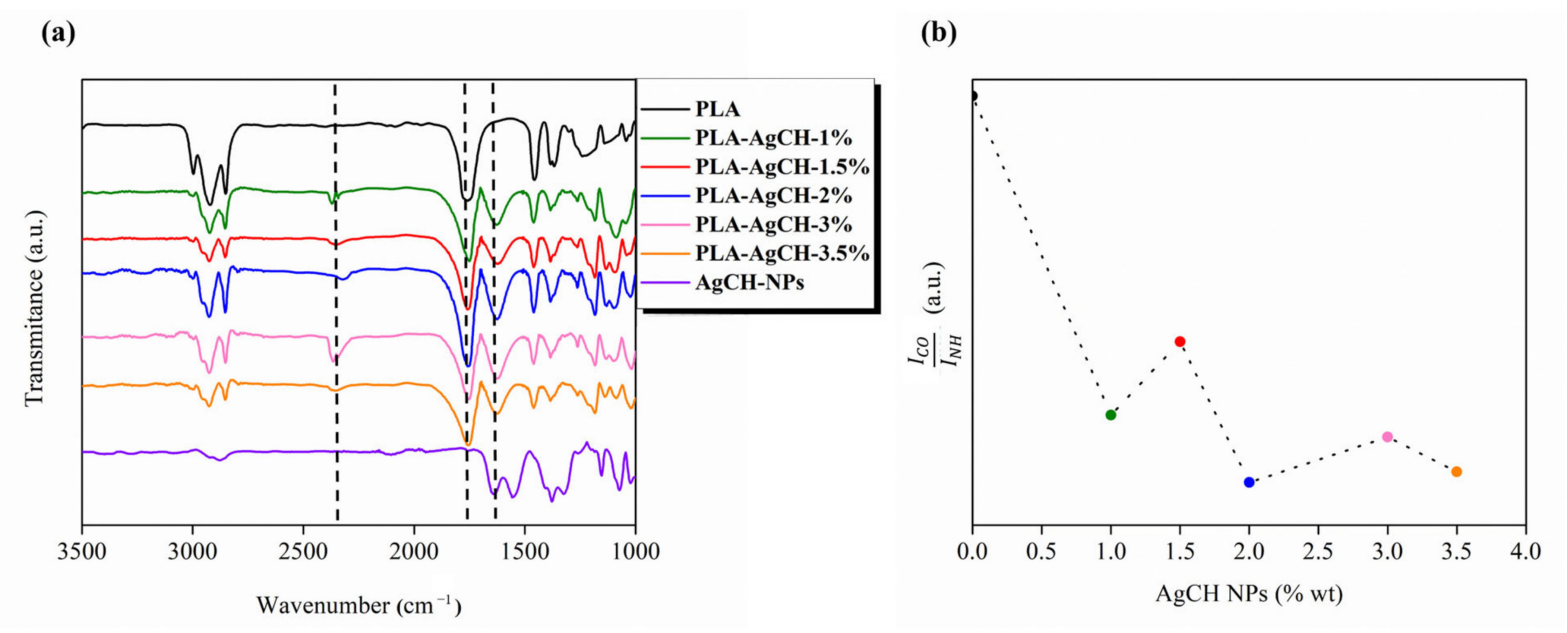
FTIR spectra of PLA, AgCH-NPs and PLA-AgCH-NPs reinforced f-fibers (**a**) and (**b**) the relation in height between the characteristic PLA peak of the carbonyl group vibration (1748 cm^−1^) to the N-H band characteristic of nanoparticle (1625 cm^−1^).

FTIR spectra of neat PLA, AgCH-NPs, and PLA-AgCH-NPs reinforced f-fibers are shown in Figure 4a. At 1748 cm^−1^ there is a carbonyl group stretching vibration band, and at 1454 cm^−1^, 1383 cm^−1^, and 1366 cm^−1^ appear -CH_3_ groups, -CH deformation, and asymmetric bands, respectively. The -C-O- stretching bands appear at 1180 cm^−1^, 1130 cm^−1^ and 1085 cm^−1^. AgCH-NPs spectrum shows the characteristics absorption bands of chitosan at 1639.49 cm^−1^ for primary amine, N-H band, 1527.62 cm^−1^ for secondary amine, N-H band, and 1388.75 cm^−1^ for methylene = CH_2_ CH_3_ band [52]. A wide band at 1625 cm^−1^ can be observed in all the reinforced f-fibers spectra, which directly demonstrates the presence of nanoparticles in all the f-fibers. This wide band can be attributed to N-H bands of nanoparticles, slightly modified by the presence of PLA. Furthermore, the difference in height between the characteristic PLA peak of the carbonyl group vibration and the N-H band characteristic of nanoparticles is represented in Figure 4b for the different NPs concentrations. It is easy to observe that, for high amounts of NPs, this difference is reduced, confirming the increased presence of nanoparticles within the f-fibers. Moreover, it is remarkable the presence of the peak at ~2300 cm^−1^, only in the reinforced f-fibers. This indicates an interaction between PLA and AgCH nanoparticles and, therefore, this peak may be due to C=O-N vibrations, which produces new symmetric and asymmetric vibrations of the COO^−^ anion [53].

**Figure 5:**
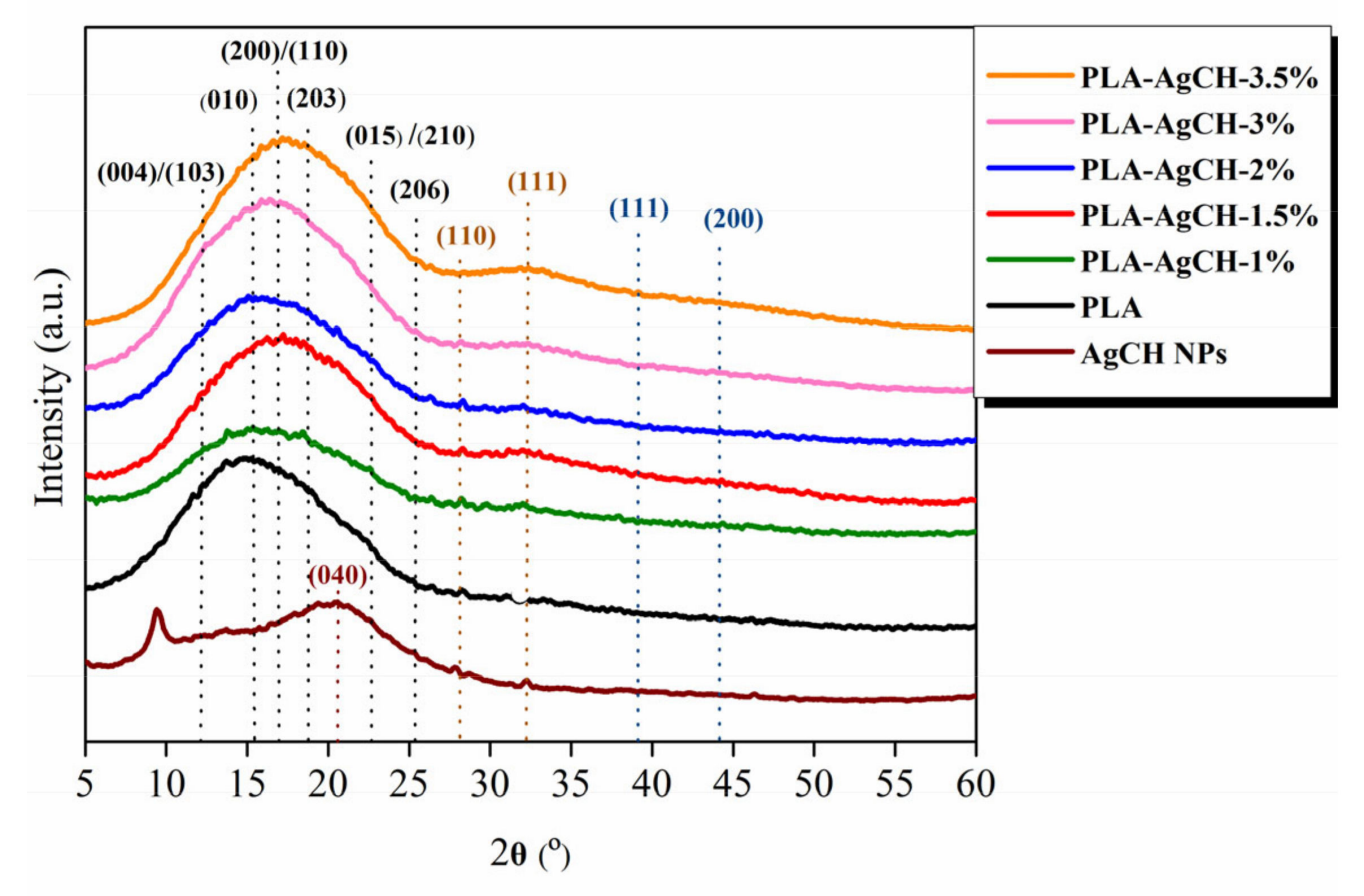
XRD spectra of PLA, AgCH-NPs and PLA-AgCH-NPs reinforced f-fibers.

In Figure 5, XRD patterns were recorded for all the samples. Neat PLA exhibits a broad reflection indicative of its amorphous nature. With the addition of nanoparticles, a slight tendency to crystallize can be observed, by the appearance of a diffraction peak at 2θ = 16.7°, corresponding to (110/200) planes of PLA, the reflection of the α-form crystals [54]. In addition, new peaks at 2θ = 15.5 ° and 2θ = 19.2° corresponding to (010) and (203) plane reflections of PLA chains belonging to α and α′ type crystals were visible, which was also consistent with the literature values [54]. This is indicative of the nanoparticles nucleating effect, and the increase of the long-range ordering of crystal packing. Apparently, if no intermolecular interactions exist between the two components, each component will form its own crystalline domains [51] and, thus, the diffraction patterns of the reinforced f-fibers mats should be the simple superposition of those of each component. The obtained results signify that two components, chitosan and PLA, have interacted with each other in a certain manner so that the original crystalline structures of each component have been disturbed or partially damaged to a different extent, leading to various crystalline structures of the reinforced f-fibers.

**Figure 6:**
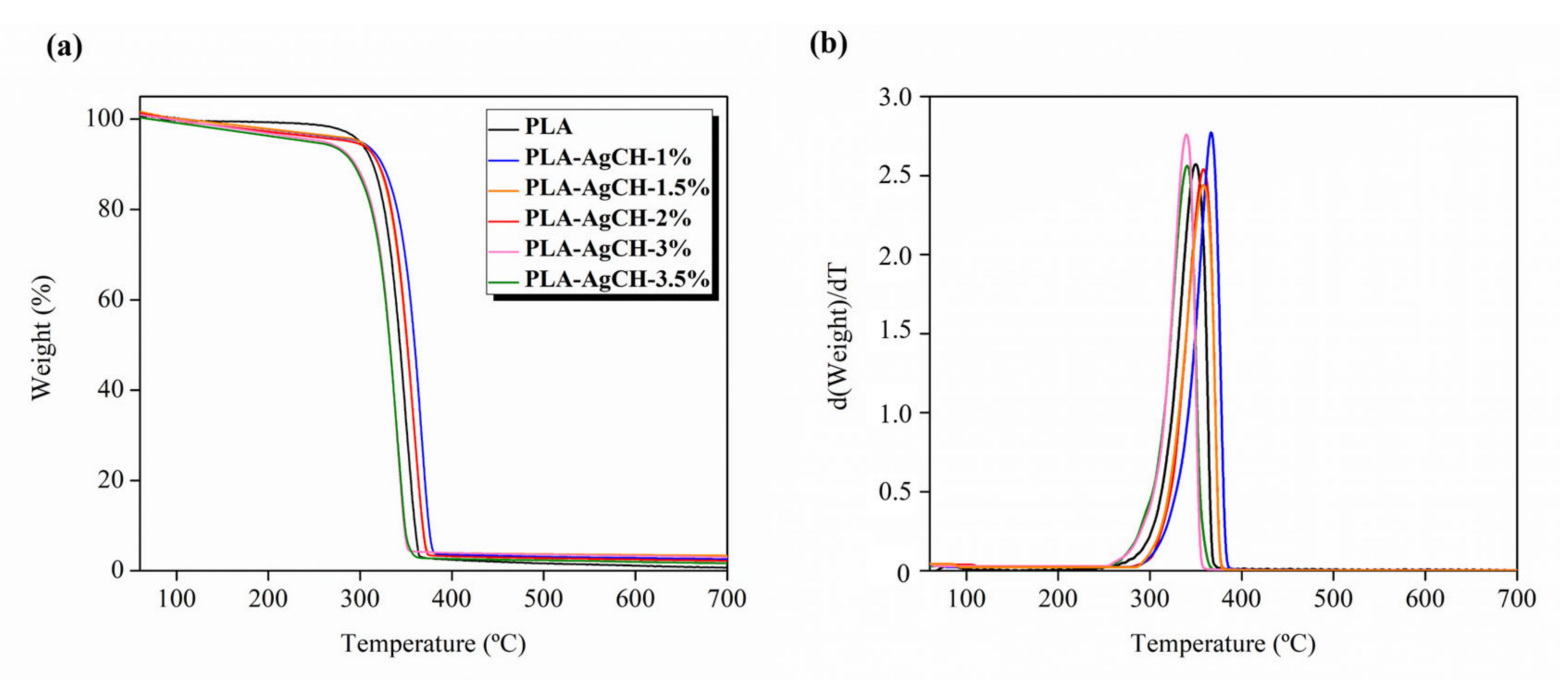
Thermogravimetric curves of neat PLA and its nanocomposites with different AgCH-NPs contents: (**a**) weight vs temperature curves, (**b**) derivative curves.

Moreover, in order to study the effect of the addition of AgCH-NPs on the thermal stability of PLA as well as on the thermal degradation mechanism, thermogravimetric analysis was conducted under nitrogen atmosphere. Figure 6 shows weight loss vs. temperature (Figure 6a) as well as their corresponding derivative curves (Figure 6b), for neat PLA and PLA-AgCH-NPs reinforced f-fibers.

Table 2 collects temperatures at 5% of weight loss for each sample as well as the maximum degradation temperature and their residue obtained at 700 °C. It can be noted that the degradation mechanism of all the samples is carried out in one step, in Figure 6b. Thus, in general, TGA results show that the addition of the different amounts of AgCH-NPs did not significantly affect the thermal degradation of the PLA matrix. It is worth noticing that there are differences in the curves at high temperatures (700 °C), due to the presence of nanoparticles that decomposed at higher temperatures. This translates into a residue that fairly matches the real amount of NPs present in each fiber formulation.

**Figure 7:**
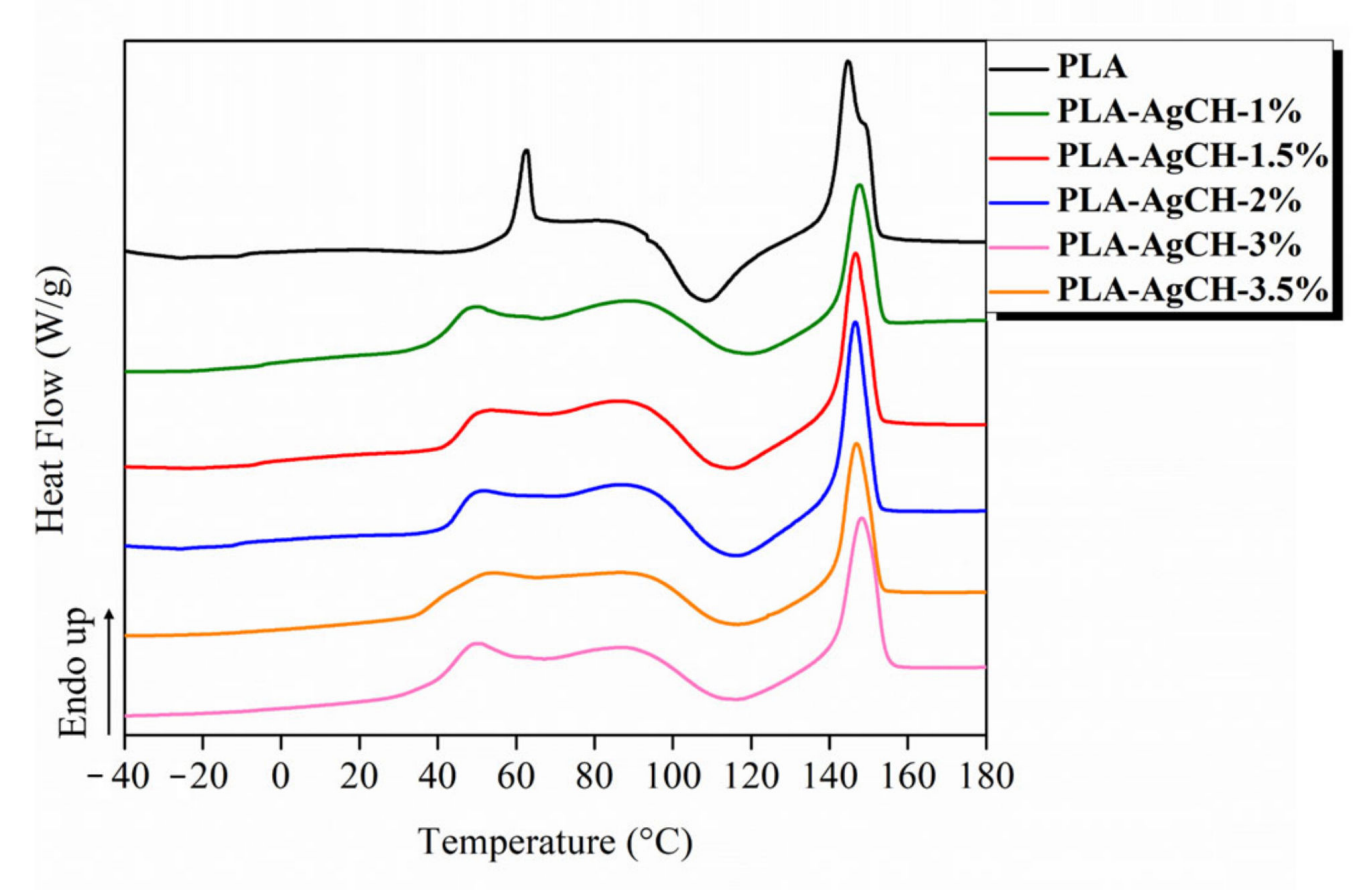
Differential scanning calorimetry (DSC) curves of PLA based f-fibers with different amount of AgCH-NPs contents.

Differential scanning calorimetry (DSC) was used to study the Tg, melting temperature, and degree of crystallization of PLA and their reinforced f-fibers. Figure 7 shows the thermograms of neat PLA and its AgCH-NPs nanocomposites f-fibers. Glass transition, melting temperatures, and the degree of crystallinity values are summarized in Table 2. The addition of AgCH-NPs into PLA resulted in significant differences over Tg values with respect to the neat matrix.

The addition of AgCH-NPs produced a decrease in Tg values from 60 °C to 38–45 °C, as indicated also in Table 2. This plasticizing effect can be explained since AgCH-NPs may occupy intermolecular spaces between polymer chains, reducing the energy for molecular motion and the formation of hydrogen bonding between the polymer chains which, in turn, increases free volume and molecular mobility. Tm values did not show notable differences when nanoparticles were present.

The characteristic mechanical behavior of all the f-fibers formulations studied is summarized in Table 3. Neat PLA showed high elastic modulus, (E), (neat PLA E = 209 MPa; AgCH-NPs nanocomposites E~1–77 MPa) compared to nanocomposites. All the nanocomposites possessed a reduction in the elastic modulus and maximum tensile strength (σmax). Elongation at break, (ε), decreases with the addition of low amounts of nanoparticles (1–1.5 wt%), and increases when higher loads are presented in the nanocomposite. The best balance of mechanical behavior in the nanocomposites was obtained for the lowest amount of AgCH-NPs tested (1 wt%) having the highest Young’s modulus and maximum tensile strength of the whole nanocomposites while retaining similar elongation at break. This sample (1 wt% of AgCH-NPs) is totally amorphous, therefore, the low amount of filler helps to retain mechanical properties closer to the neat PLA matrix that also possesses the lowest crystallinity. Thus, it seems that the nanoparticles did not disturb the chain mobility, allowing for an increased elongation at break, which is also enhanced.

Once the mechanical and thermal characterizations of the materials were carried out and in order to evaluate the thermally-activated shape-memory response of the material, three thermo-mechanical cycles were performed by DMA analysis for each formulation at 60 °C. In our previous work, the thermally-activated shape-memory response of woven non-woven PLA fibers obtained by a similar processing method based on the electrospinning technique was successfully demonstrated at 60 °C [13]. Thus, the shape-memory capability of fibers obtained by the force-spinning technique will be studied in this section in order to evaluate the suitability of the force-spinning processing technique for producing smart materials.

**Figure 8:**
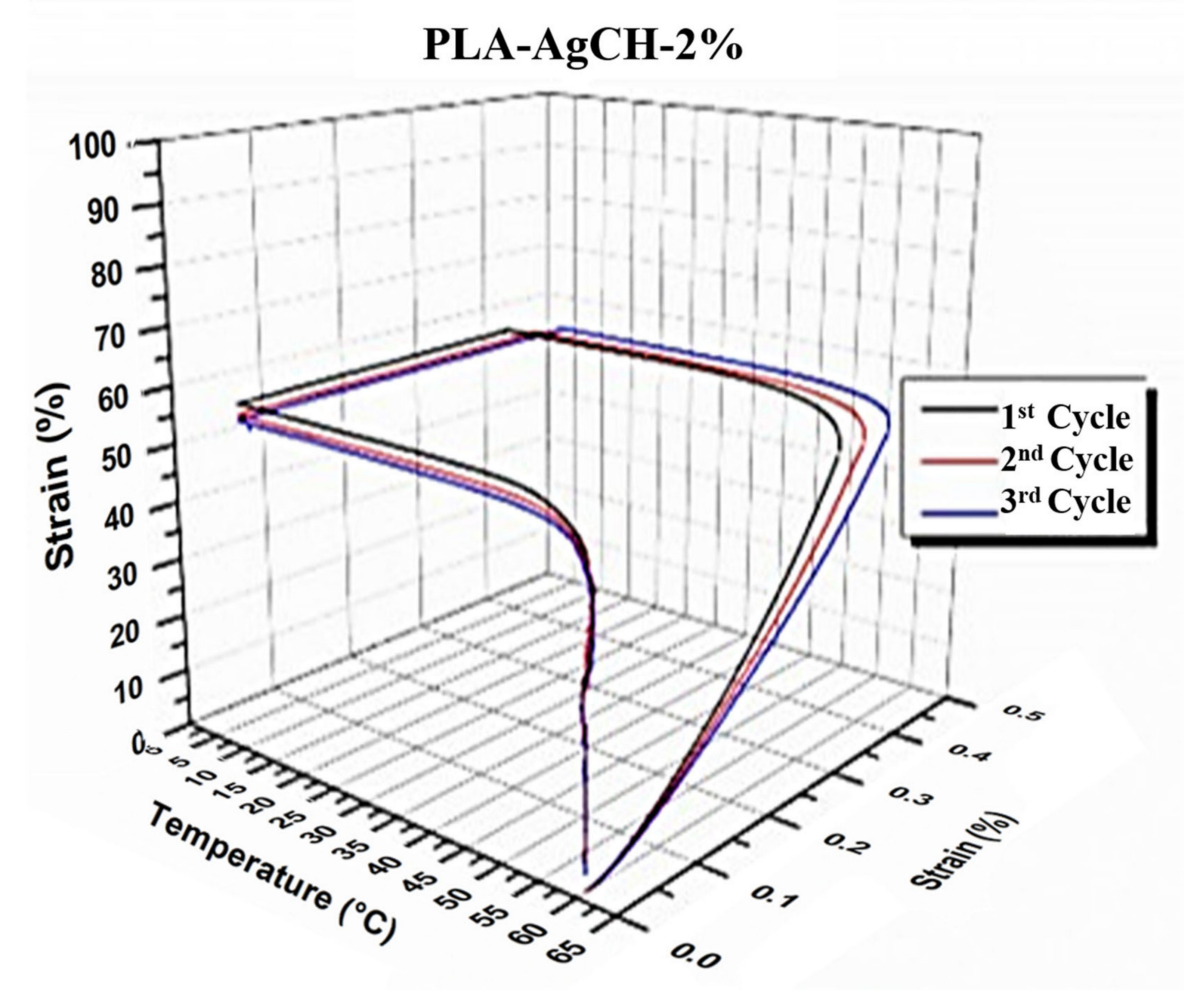
3D thermo-mechanical cycles performed at 60 °C for PLA-AgCH-2%.

As a preliminary study, we verify that reinforced f-fibers are able to show thermally-activated shape-memory response, activated by Tg, which means that we studied how the addition of the higher amount of nanoparticles, that is 2 wt% AgCH NPs, affects the shape-memory behavior. With this aim, the thermo-mechanical cycles for the reinforced formulation are reported in Figure 8. In particular, we obtain an important result, that is PLA f-fibers reinforced with 2 wt% AgCH NPs show thermally-activated shape-memory response at 60 °C. In this case, adding 2 wt% AgCH NPs successfully enhances the ability to fix the temporary form by obtaining values of higher than 80% for R_f_ during all the thermo-mechanical cycles.

Additionally, good recovery ratios were reported for each thermo-mechanical cycle showing the excellent shape recovery of the original shape at 60 °C during all the tests performed with R_r_ values higher than 90% for all the thermo-mechanical cycles.

Looking at the results obtained by TGA analysis, the degradation temperature obtained for this formulation was 367 °C (the highest of all the formulation tested) which translates into greater compatibility between nanofillers and the polymeric matrix. This good interaction between AgCH-NPs and PLA matrix improves the fixing capacity of the fibers while the recoverability of the original shape is preserved, showing recovery ratios higher than 90% in all the thermo-mechanical cycles.

**Figure 9:**
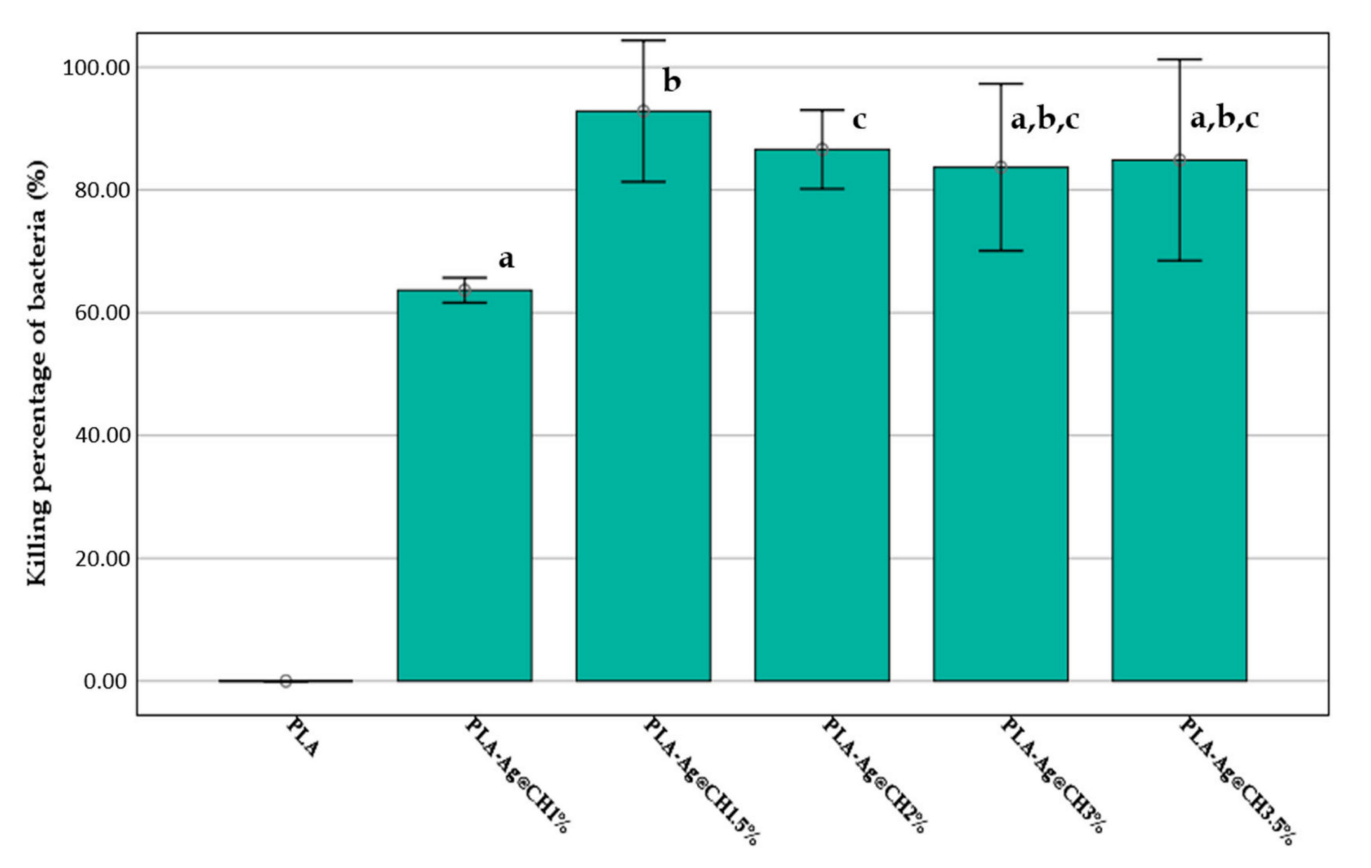
Percentage of killing bacteria for the different nanocomposites. Values having the same letter are not significantly different for the Tukey test (significance level of *p* ≤ 0.05).

The effectiveness of these nanocomposites against gram-positive *S. aureus* bacteria was evaluated by the ASTM standard method [45]. Figure 9 displays the antibacterial activity of all nanocomposites represented as the percentage of bacteria kill and their confidence interval of 95%, which was calculated by the difference between the CFU after contact with control substrates (PLA and none) and CFUs after contact with the polymeric nanocomposites. When AgCH-NPs are introduced, this confers antibacterial activity to the matrix. The behavior against gram-positive bacteria is very effective in fibers since with 1 wt% of nanoparticles, the killing percentage of bacteria reaches 65% and from 1.5 wt%, this percentage becomes 90%.

**Figure 10:**
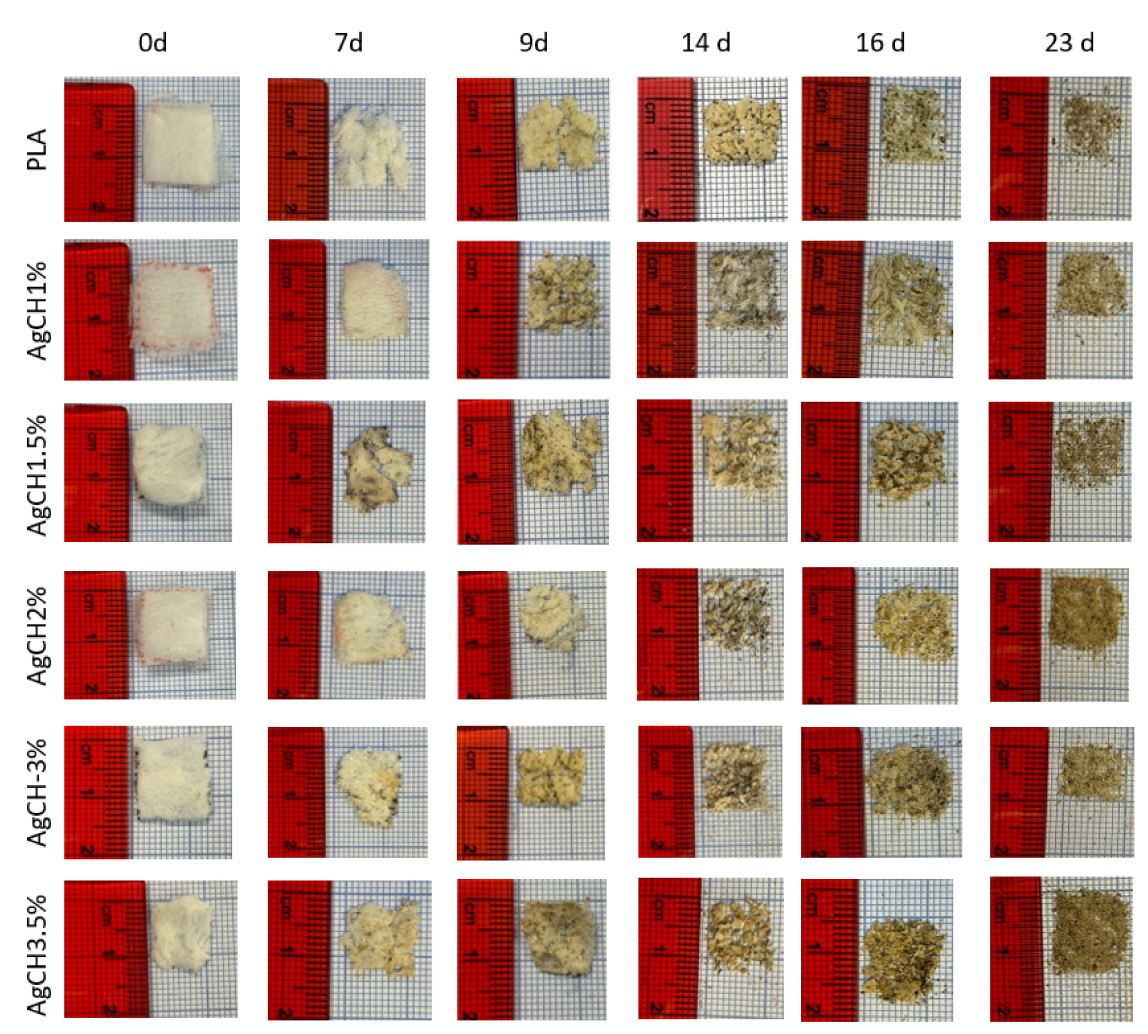
Disintegrated samples under composting conditions.

In order to evaluate the ability of PLA and PLA-AgCH-NPs f-fibers to undergo disintegration, firstly, a visual examination of samples at different times when subjected to composting conditions was performed and results are collected in Figure 10. It should be taken into account that the degradation experiments took place at 58 °C, which is around the T_g_ of PLA and higher than the T_g_ of the nanocomposite f-fibers. The degradation rate is much greater above the glass transition temperature as polymer chains become more flexible and water absorption increases, accelerating hydrolysis and microbial attachment. PLA is susceptible to hydrolysis due to the hydrolyzable functional groups in its backbone. On the seventh day, fragmentation and weight loss of the composites were already observed for all samples, especially in low-load fibers.

The environmental degradation process of PLA is affected by its material properties such as molecular first-order structural (molecular weight, optical purity) and higher order structures (crystallinity, T_g,_ and T_m_), and by environmental factors such as humidity, temperature, and catalytic species (pH and the presence of enzymes or microorganisms) [55]. Crystalline regions hydrolyze much more slowly than the amorphous regions as water diffuses more readily into the less organized amorphous regions compared to the more ordered crystalline regions, causing greater rates of hydrolysis and increased susceptibility to biodegradation [46]. This explains the results obtained in Figure 10, the faster degradation of PLA and PLA AgCH 1–2%, which are less crystalline than PLA AgCH 3–3.5%, as we have seen in the DSC analysis.

## 4. Conclusions

In this work, PLA-based fiber mats, containing between 0 and 3.5% by weight of chitosan-functionalized silver nanoparticles obtained through a green protocol, have been prepared by means of centrifugal force-spinning. Their morphological, thermal, and mechanical properties have been profusely characterized, as well as their functional properties in terms of biodegradation, antibacterial and smart properties. Homogeneous fiber mats with average fiber diameters in the micrometer scale have been prepared. We found that the best balance in terms of mechanical behavior was obtained for the lowest amount of AgCH-NPs, that is 1 wt%, which presented the highest Young’s modulus. Moreover, with 2 wt% of AgCH-NPs, we obtain a very good thermally-activated shape-memory effect with very high values for Rf and Rr. Furthermore, AgCH-NPs confer antibacterial activity to the matrix. In particular, the behavior against gram-positive bacteria is very effective in fibers, since with 1 wt% of nanoparticles, the killing percentage of bacteria reaches 65%, and from 1.5 wt%, this percentage becomes 90%. Finally, all the samples are disintegrable under composting conditions, conferring promising properties for possible food packaging application and in the biomedical field.

## Figures and Tables

**Table 1:** PLA-AgCH-NPs formulations.

Samples	PLA (wt%)	AgCH-NPs (wt%)
PLA	100	0
PLA-AgCH-1%	99	1
PLA-AgCH-1.5%	98.5	1.5
PLA-AgCH-2%	98	2
PLA-AgCH-3%	97	3
PLA-AgCH-3.5%	96.5	3.5

**Table 2:** Temperatures at different weight losses of neat PLA and the different formulations containing AgCH-NPs.

Samples	T_max_	T_5_	T_700_	Tg (°C)	Tm (°C)	Xc (%)
PLA	350	300	0.73	60	145	0.29
PLA AgCH1%	341	246	1.67	41	148	4.20
PLA AgCH1.5%	357	288	2.22	46	147	6.31
PLA AgCH2%	367	298	2.56	45	146	7.61
PLA AgCH3%	340	262	3.23	44	148	8.05
PLA AgCH3.5%	363	297	3.67	38	147	9.96

**Table 3:** Mechanical properties for neat PLA and for formulations of PLA containing AgCH-NPs.

Sample	E (MPa)	σ (MPa)	ε Break (%)
PLA	209.07 ± 46.71 ^a^	9.51 ± 1.79 ^a^	15.79 ± 8.89 ^a,b^
PLA-AgCH 1%	76.73 ± 18.14 ^b^	3.32 ± 0.89 ^b^	11.31 ± 1.69 ^a,b^
PLA-AgCH 1.5%	43.98 ± 15.89 ^b^	1.34 ± 0.47 ^b^	5.92 ± 1.74 ^a^
PLA-AgCH 2%	35.32 ± 6.79 ^b^	1.67 ± 0.74 ^b^	10.04 ± 3.55 ^a^
F ratio	14.02	14.39	3.69
*p*-Value	0.0000 *	0.0000 *	0.0150 *

Different letters in the column indicate significant differences according to Tukey’s test (*p* < 0.05). * Values are significant at *p* < 0.05.

## Data Availability

The data presented in this study are available on request from the corresponding author.
